# Effects of health residency programs on services and care networks of the Brazilian Unified Health System: rapid scoping review, Brasília, 2025

**DOI:** 10.1590/S2237-96222026v35e20250464.en

**Published:** 2026-02-26

**Authors:** Pedro Emanuel do Nascimento Fernandes, Ana Gabriela Costa Normando, Keitty Regina Cordeiro de Andrade, Fabianne de Castro Teixeira, Priscilla Azevedo Souza, Paloma Ribeiro Pires Simas, Denise Fernandes Leite, Fernando Canto Michelotti, Michele Sacramento dos Santos, Fernanda Madeira de Ley Botelho Cunha, Marta Roberta Santana Coelho, Fábio Henrique Cavalcanti de Oliveira

**Affiliations:** 1 Ministério da Saúde, Departamento de Ciência e Tecnologia, Brasília, DF, Brazil Ministério da Saúde Departamento de Ciência e Tecnologia Brasília DF Brazil; 2 Ministério da Saúde, Departamento de Gestão da Educação na Saúde, Brasília, DF, Brazil Ministério da Saúde Departamento de Gestão da Educação na Saúde Brasília DF Brazil

**Keywords:** Internship and Residency, Health Systems, Health Services, Scoping Review, Brazil, Internado y Residencia, Sistemas de Salud, Servicios de Salud, Revisión de Alcance, Brasil

## Abstract

**Objective:**

To investigate the effects of residency programs on services and care networks of the Brazilian Unified Health System (*Sistema Único de Saúde*, SUS).

**Methods:**

This rapid scoping review was conducted in accordance with the Joanna Briggs Institute and the Preferred Reporting Items for Systematic Reviews and Meta-Analyses extension for Scoping Reviews checklist. Searches were conducted in January 2025, with no time restriction, across the PubMed, Web of Science, and Virtual Health Library databases, as well as gray literature via Google Scholar. Study selection was performed using the Rayyan tool, including full-text articles in English, Portuguese, and Spanish. The extracted data were analyzed using a narrative approach.

**Results:**

Of the 2,563 studies identified, 12 articles were included, with publications between 2012 and 2024. The studies examined residency programs across Brazil, with particular emphasis on the Northeast, including multiprofessional, medical, and family and community medicine residencies. The synthesis of results was organized into five axes: (i) service resolvability; (ii) quality of care; (iii) integration between education and service; (iv) improvement of care; and (v) dissemination of good practices. Positive impacts of residency programs included expanding access, enhancing clinical qualifications, and fostering interprofessional and collaborative practices. Education-service integration strengthened resolvability by reorganizing work processes, stimulating critical reflection, and aligning professional practice with the principles of the SUS. The presence of residents fostered innovations, strengthened bonds with users, and promoted changes in care delivery.

**Conclusion:**

Residency programs contribute to improving care within the SUS by promoting access, resolvability, quality, collaborative practices, and innovation, highlighting their structuring role within care networks.

## Resumen

**Objetivo:**

Investigar los efectos de los programas de residencia en los servicios y las redes de atención del Sistema Único de Salud (SUS).

**Métodos:**

Se realizó una revisión de alcance rápida siguiendo las recomendaciones del Instituto Joanna Briggs y la lista de verificación *Preferred Reporting Items for Systematic Reviews and Meta-Analysis extension for Scoping Reviews*. Las búsquedas se llevaron a cabo en enero de 2025, sin restricción temporal, en las bases de datos PubMed, Web of Science y Biblioteca Virtual en Salud, así como en la literatura gris a través de Google Scholar. La selección se realizó mediante la herramienta Rayyan, incluyendo artículos completos en inglés, portugués y español. Los datos extraídos se analizaron mediante un enfoque narrativo.

**Resultados:**

De 2.563 estudios identificados, se incluyeron 12 artículos publicados entre 2012 y 2024. Los estudios abordaron programas de residencia en diferentes regiones de Brasil, con énfasis en el Nordeste, incluyendo residencias multiprofesionales, médicas y de medicina familiar y comunitaria. La síntesis de los resultados se organizó en cinco ejes: (i) capacidad de resolución de los servicios; (ii) calidad de la atención; (iii) integración entre la enseñanza y el servicio; (iv) mejora de la asistencia; y (v) difusión de buenas prácticas. Se evidenciaron impactos positivos de las residencias en la ampliación del acceso, la mejora de la calificación clínica y la inducción de prácticas interprofesionales y colaborativas. La integración entre la enseñanza y el servicio fortaleció la capacidad de resolución al reorganizar los procesos de trabajo, estimular la reflexión crítica y alinear la actuación con los principios del SUS. La presencia de residentes impulsó innovaciones, fortaleció los vínculos con los usuarios y promovió transformaciones en el ámbito asistencial.

**Conclusión:**

Las residencias contribuyen a calificar la atención en el SUS al promover el acceso, la capacidad de resolución, la calidad, las prácticas colaborativas y la innovación, evidenciando su papel estructurante en las redes de atención.

**Ethical aspects**

This research used public domain anonymized databases.

## Introduction 

The training of qualified professionals is one of the fundamental pillars for consolidating the Brazilian Unified Health System (*Sistema Único de Saúde*, SUS), as it structures a workforce capable of responding to the population’s health needs with quality, comprehensiveness, and equity ^1^. In this context, health residency programs (medical, multiprofessional, or uniprofessional) have emerged as powerful strategies for continuing education and professional qualification by promoting the direct insertion of professionals in training into SUS services ^2^^,^^3^.

Established as postgraduate specializations, residencies in the health professional field are characterized primarily by in-service training, grounded in the principles of the SUS and aimed at working with users, families, and communities in their real-life contexts ^4^. This practical immersion provides critical, situated training that articulates technical-scientific knowledge with the realities of communities. It enables professionals to develop clinical, managerial, ethical, and political-institutional competencies in constant dialogue with the challenges of health services ^5^.

The presence of residents in public services has the potential to stimulate organizational transformations, promoting the adoption of evidence-based practices, the qualification of care processes, and the expansion of the teams’ response capacity ^6^. Furthermore, it may facilitate workforce renewal by continuously updating clinical practice and incorporating recent technical and political guidelines, thereby consolidating a culture of continuous education ^6^^,^^7^.

Health residencies are positioned as a formative strategy to expand vacancies in priority areas, enhance the teaching-care role, promote citizenship, and foster interprofessional collaboration within services ^6^. These residencies aim to qualify training by focusing on competencies aligned with community needs, addressing structural discrimination, promoting user-centered care, and strengthening the integration among education, service, and community ^8^.

At the same time, residency programs have also faced criticism for structural and organizational weaknesses in practice settings, often marked by infrastructure deficits, scarcity of material and human resources, team overload, and inadequate pedagogical planning ^2^. These issues directly affect preceptorship and mentorship, particularly due to the precarious nature of the preceptor role, which undermines the effective supervision of residents ^9^

In recent years, concern has also emerged regarding residents’ mental health conditions, as they are frequently exposed to exhausting schedules, situations of institutional violence and harassment, lack of psychosocial support, and precarious employment relationships ^10^. Considering this overall context, the objective of this study was to investigate the effects of residency programs on SUS services and care networks.

## Methods 

### Study design

This is a rapid scoping review, which employs methodological shortcuts in the execution of certain steps of a traditional scoping review. Such shortcuts reduce the time required for its development. 

This rapid scoping review was conducted in accordance with the methodology outlined in the Reviewer’s Manual for Scoping Reviews by the Joanna Briggs Institute ^11^. The review was reported in accordance with the Preferred Reporting Items for Systematic Reviews and Meta-Analyses extension for Scoping Reviews (PRISMA-ScR) guidelines ^12^.

The research question guiding this review was: “What are the effects of health residency programs (medical, multiprofessional, or uniprofessional) on the quality and/or problem-solving capacity of services and care networks within the Brazilian Unified Health System (SUS)?” This question was developed based on the PCC (population, concept, and context) acronym, as follows: 

Population: health residency programs. 

Concept: effects on the quality and resolvability of services and care networks. 

Context: Brazilian Unified Health System (SUS). 

### Eligibility criteria

The following were included: (i) studies on the effects of residency programs on quality, resolvability, and outcomes in services of the Brazilian public health system; (ii) methodological designs such as observational studies (cross-sectional, cohort, or case-control), program or policy evaluation and/or implementation studies, exploratory-descriptive studies with qualitative, quantitative, or mixed approaches, as well as systematic, integrative, or scoping reviews; (iii) institutional documents presenting systematized data on the effects of residencies in health services; and (iv) publications in Portuguese, English, or Spanish, regardless of the year of publication. The following were excluded: (i) studies that did not address health residency programs linked to public health systems; (ii) narrative reviews, document analyses, institutional documents without empirical data, conceptual essays, or experience reports; (iii) studies that did not present data related to the quality and/or resolvability of services or care networks; (iv) publications available only as conference abstracts, editorials, commentaries, or expert opinions; and (v) studies not available in full text.

### Information sources 

Searches were conducted in the Medline (via PubMed), Web of Science, and Virtual Health Library databases. In addition, searches were conducted in Google Scholar, encompassing both peer-reviewed articles and gray literature (theses and dissertations). 

Manual searches of the references of relevant publications to identify additional potentially eligible studies were not conducted, considering the rapid scoping review nature of this study. No restrictions were applied regarding language or publication status.

### Search strategy 

A pilot search strategy was developed for the Medline database (via PubMed) and adapted for the other databases and for Google Scholar. The final strategy for each database was presented (Supplementary Table 1). Search results were imported into Rayyan software ^13^ to remove duplicates and proceed with the review steps. Searches in all databases were conducted on 15 January 2025.

### Selection process 

Three researchers (PENF, AGCN, and KRCA) independently selected studies, unpaired and unblinded, based on titles and abstracts using the Rayyan platform ^13^. In Google Scholar, the first 100 publications identified and ordered by relevance were considered in this initial stage. Subsequently, the full texts of eligible studies were analyzed using a pretested and calibrated electronic spreadsheet. Any discrepancies or doubts were resolved by consensus in a joint meeting of the three researchers.

### Data extraction process and items

Three researchers (PENF, AGCN, and KRCA) independently extracted data using a standardized, pretested electronic spreadsheet. The process was calibrated in an initial meeting.

### Data items

Data on the general characteristics of residency programs and health services were extracted as described below.

General characteristics: author, year, location, institution, study design, and study period. The participant profile (users, residents, preceptors, tutors, coordinators, managers, and workers from the SUS or other public systems), the type of health service, and its relation to the system were also considered.

Residency program characteristics: residency modality (medical, multiprofessional, or uniprofessional), professional category, specialty, field of practice, geographic area (urban, rural, or priority), duration, and workload.

Health service characteristics: quality indicators (user satisfaction, accessibility, continuity, and care coordination) and resolvability indicators (queue reduction, impact on indicators, problem-solving). Results related to education-service integration, promotion of scientific production and good practices, professional qualification, recognition of preceptorship, and reduction of regional inequalities were also analyzed.

### Critical appraisal of individual evidence sources

In this study, a risk-of-bias assessment of the included studies was not performed.

### Data synthesis 

Extracted data were synthesized based on study results and organized into five thematic axes, defined according to the recurrence and relevance of the information: (i) service resolvability; (ii) quality of health care; (iii) education-service integration; (iv) care improvement; and (v) dissemination of good health practices. Findings were presented narratively, and no meta-analysis was conducted because the data were qualitative. The preparation and discussion of results were carried out by all authors (PENF, AGCN, and KRCA).

**Table 1:** Characteristics of the included studies (n=12). Brasília, 2025

Author, year of publication	Study characterization		Population characteristics	Residency program characterization	
	Study design	Location	Participants	Residency program modality	Level of patient care
Qualitative studies					
Barreto, 2019	Systematization of experience	Paraíba, Brazil	Professors and researchers	Medical residency in family and community medicine	Primary health care
Fernandes, 2015	Social representation	Ceará, Brazil	Health professionals	Medical residency in anesthesiology	Tertiary health care
Landim, 2012	Theoretical-methodological framework in phenomenological research	São Paulo, Brazil	Nursing residents	Multiprofessional residency program in family health	Primary health care
Machado, 2018	Exploratory descriptive research	Ceará, Brazil	Residents in nursing, psychology, social work, and physical education	Integrated residency with emphasis on collective mental health, family and community health, and public health	Primary health care
Pinheiro, 2023	Exploratory descriptive research	Ceará, Brazil	Nursing residents	Multiprofessional residency in family and community health	Primary health care
Pinto, 2022	Exploratory descriptive research	Rio Grande do Norte and São Paulo, Brazil	Coordinators and residents	Multiprofessional health residency programs	Primary health care
Santos, 2024	Documentary research and interviews	Pernambuco, Brazil	Managers, preceptors, residents, and social control	State policy for health professional residency in Pernambuco	All levels of care
Souza, 2022	Qualitative case study	Pernambuco, Brazil	Managers, preceptors, residents, and users in *quilombola* communities (self-identified Afro-Brazilian traditional communities)	Multiprofessional health residency	Primary health care
Quantitative studies					
Christofoletti, 2015	Cross-sectional study	Mato Grosso do Sul, Brazil	Users	Multiprofessional health residency	Tertiary health care
Costa, 2021	Cross-sectional study	Ceará, Brazil	Users	Medical residency in family and community medicine	Primary health care
Jantsch, 2023	Retrospective longitudinal analysis	Rio de Janeiro, Brazil	Users	Medical residency in family and community medicine	Primary health care
Mixed-methods studies (qualitative and quantitative)					
Santos, 2023	Mixed-methods case study	Pernambuco, Brazil	Health residency governance actors	Uniprofessional and multiprofessional health residencies	All levels of care

## Results 

The structured electronic searches identified 2,563 studies. After removing duplicates, 2,423 studies proceeded to title and abstract screening. Of these, 60 studies were selected for full-text reading based on the previously established eligibility criteria. At the end of this stage, 12 studies met the criteria and were included in the review ^14^^-^^25^. The 48 excluded studies, along with the reasons for exclusion, are detailed in Supplementary Table 2. The study selection process is described in Figure 1. The theses and dissertations identified did not meet the eligibility criteria and were excluded at both the initial screening and full-text evaluation stages.

A total of 12 studies were included ^14^^-^^25^, comprising 8 qualitative studies ^14^^,^^17^^,^^19^^-^^22^^,^^24^^,^^25^, 3 quantitative studies ^15^^,^^16^^,^^18^, and 1 mixed-methods study (qualitative and quantitative) ^23^. The publications, published between 2012 and 2024, covered residency programs implemented in various regions of Brazil. The Northeast region was the most represented, with emphasis on Ceará, which accounted for 4 studies ^16^^,^^17^^,^^20^^,^^21^, followed by Pernambuco with 3 studies ^23^^-^^25^. 

**Figure 1:**
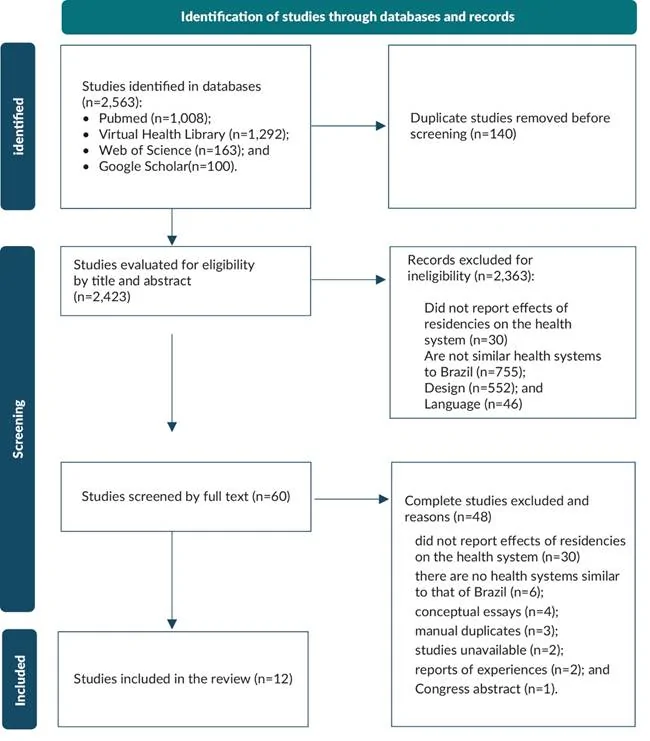
Study selection and inclusion process in the scoping review. Brasília, 2025

In the identified studies, all residency programs took place in public institutions. Most investigated multiprofessional health residency programs involve professionals from nursing, psychology, physiotherapy, speech therapy, dentistry, nutrition, pharmacy, physical education, and social work ^15^^,^^19^^-^^25^. Studies on medical residency were also included ^17^, particularly those focused on family and community medicine ^14^^,^^16^^,^^18^. 

Regarding service placement, most evaluated programs were linked to primary health care ^14^^,^^16^^,^^18^^-^^22^^,^^25^. Two studies investigated programs within tertiary care services ^15^^,^^17^, and 2 covered programs across different levels of care ^23^^,^^24^. Details of study characteristics, including location, residency program, and level of care, were presented (Table 1).

The synthesis of results was organized based on the most recurring themes identified in the primary studies. The theme of service resolvability was addressed in 6 studies ^2^^,^^15^^,^^16^^,^^18^^,^^23^^,^^25^; quality of health care in 5 ^16^^,^^17^^,^^19^^,^^22^^,^^24^; education-service integration in 8 ^2^^,^^15^^,^^19^^-^^23^^,^^25^; care improvement in 2 ^16^^,^^24^; and dissemination of good health practices in 4 ^17^^,^^21^^,^^22^^,^^24^. Based on this thematic frequency and relevance, the findings were categorized into 5 analytical axes that structured the presentation of results: (i) service resolvability; (ii) quality of health care; (iii) education-service integration; (iv) care improvement; and (v) dissemination of good health practices. The specific contributions of each included study, by thematic axis, are available in Supplementary Table 3.

The studies analyzed showed that health residency programs positively affect the resolvability of SUS services ^25^. Notable effects included increased access, reduced avoidable hospitalizations, and greater integration between levels of care ^15^^,^^18^. These effects enhanced the quality of care and strengthened health care networks ^14^. The expansion of medical residency in family and community medicine was highlighted as a key factor in increasing resolvability in primary health care ^25^. The increased presence of family physicians in basic health units improved service coverage, problem-solving capacity, and municipal involvement in medical training and care management ^14^.

Quantitative studies reinforced this impact by showing that physicians trained in family and community medicine residencies are associated with reduced hospitalizations for conditions sensitive to primary care. Patients followed by these professionals had a lower risk of hospitalization for chronic diseases, including hypertension, diabetes, cardiovascular, and respiratory conditions, demonstrating the effectiveness of primary health care in preventing complications and hospital admissions ^15^^,^^18^.

Multiprofessional residents in primary health care units have enhanced local resolvability through active outreach, strengthened bonds, and coordination among professionals and services within the care network. The perceptions of managers, preceptors, and users were largely favorable, highlighting service improvements, greater accessibility, and a reduced need for referrals ^24^^,^^25^.

The presence of residents in health services has been a relevant factor in improving the quality of care by promoting reflective practices, interdisciplinary integration, and expanded access ^17^^,^^22^. Residents’ practice encourages knowledge exchange, reorganization of work processes, and strengthening of health care networks ^24^. 

In tertiary care, medical residency in anesthesiology contributed to concrete changes in care practices, such as the adoption of preanesthetic visits, which had previously been nonexistent. This improved perioperative care and strengthened integration among different professional categories. The presence of residents prompted critical reflection among preceptors and led to improvements in user safety ^17^.

In multiprofessional health residency programs, residents’ reports highlighted the programs’ capacity to integrate diverse services and strengthen health training within the SUS, with direct impacts on team qualification and the consolidation of health care networks. In particular, hospital-based experiences undertaken by nurses during residency were later applied in primary health care, thereby positively influencing team practices and promoting opportunities for ongoing education ^22^.

The evaluation of the state residency policy in Pernambuco also showed immediate gains in the quality of care, particularly due to the increase in the number of specialized professionals in the services. This resulted in greater user recognition and expanded access to SUS actions ^23^^,^^24^.

Education-service integration has emerged as a central component of health residency programs, facilitating the articulation between theory and practice, strengthening professional training, and improving services within the SUS ^25^. The insertion of residents into care settings contributes to the reorganization of care processes, stimulates critical reflection, and enhances continuing education among teams.

Residency provides formative experiences that complement gaps left by undergraduate education by bringing residents closer to service realities and SUS guidelines ^14^. The diversity of settings and practices, including hospital and primary health care experiences, supports the development of technical, ethical, and relational competencies within a continuous-learning perspective ^21^.

The presence of residents has also driven innovation in services by encouraging collaborative practices, promoting collective knowledge construction, and strengthening interaction among professionals, managers, and training institutions ^22^. Joint practice has been recognized as a factor in transforming institutional culture and care quality.

However, significant challenges were also identified, including weak coordination between educational institutions and health services, a lack of systematic pedagogical support, and precarious working conditions for tutoring and preceptorship roles ^23^. Criticism was raised regarding the lack of alignment between curricular content and the realities residents experience in services ^20^. 

The integration of residents into health services has contributed to improvements in care by bringing professional training closer to territorial realities and SUS practices ^16^. This immersion improves clinical practice, expands service provision, strengthens team decision-making, and develops technical, interpersonal, and communication skills ^19^. Coexistence between residents and professionals also facilitates the overcoming of practices centered on the biomedical model, aligning care more closely with comprehensive care guidelines ^22^.

User perceptions of primary health care were assessed using the Primary Care Assessment Tool, which found that the presence of residency programs, especially in family and community medicine, positively affected service evaluations ^16^. This factor increased the overall quality scores assigned by users ^16^.

In the hospital context, nursing residents reported clinical advances, a more critical perspective, and greater sensitivity to users’ needs ^19^. Knowledge exchange between residents and teams has enabled more integrated care models aligned with SUS guidelines and the realities of primary health care ^22^. The presence of residents contributes to rethinking disease-centered, fragmented practices and promotes broader, person-centered approaches ^22^.

Good health practices arise from interprofessional collaboration and team integration, representing one of the main indirect effects of residents’ practice. It was observed that the presence of these professionals enhanced communication and cooperation among anesthesiologists, surgeons, and nursing teams, thereby promoting shared decision-making and reducing conflicts in the operating room ^17^. 

Experience in the SUS helped challenge prejudices about work in primary health care ^17^^,^^22^. Residency programs, by integrating education and care, foster critical and reflective training on the realities of the SUS, thereby developing professionals with critical thinking, technical competence, and a commitment to system transformation ^21^^,^^24^.

Hospital experience was also decisive in developing technical skills and clinical judgment. Immersion in care during this period provided residents with a better understanding of health conditions and improved essential competencies, such as decision-making ^19^. This continuous learning process is fundamental to the training of professionals capable of addressing public health challenges ^19^.

## Discussion 

The results of this review indicate the strategic role of health residency programs in improving the quality of care delivered within the SUS, particularly by fostering collaborative practices, promoting the reorganization of work processes, and contributing to the management of health conditions across different levels of care. Residency programs were observed to act as important drivers of service resolvability, strengthening bonds with the community, revising clinical practices, and promoting interprofessional approaches. This favors team integration and positively influences care flows ^26^. 

Despite the advances identified, significant challenges persist. This review included studies published between 2012 and 2024, encompassing diverse designs and populations, and revealed a substantial gap in the measurement of the systemic effects of residency programs. A predominance of qualitative approaches was observed, generally focusing on local contexts, pedagogical analyses, or experience reports from individuals affiliated with or previously involved in the programs (residents, preceptors, tutors, or faculty members). 

Although these studies contributed to documenting experiences, practice contexts, and training processes, they offered limited potential for extrapolation. They did not advance toward a broader assessment of the effects of residencies on the health system. Therefore, a gap remains in the literature for primary studies with greater methodological rigor that can systematically and comprehensively investigate the contributions of these programs to services and care networks.

As this is a rapid scoping review, methodological limitations inherent to the design must be considered, including the use of shortcuts in study selection and data extraction, conducted in an unblinded, unpaired manner, as well as the absence of manual searches to identify potentially eligible studies. Although the research team previously calibrated these procedures, they do not eliminate the risk of selection and analysis bias.

In many contexts, improvements in care and reorganization of work processes observed during the residency period tend to dissipate after residents leave the practice setting. This indicates fragility in the institutionalization of practices and in the consolidation of structural changes ^27^. In several situations, residents are allocated to meet service demands and assume responsibilities that sometimes exceed expectations for their stage of professional training ^28^. This reality may weaken the formative character of residency programs ^29^.

Many residents reported challenges, including intense workloads, the accumulation of care-related activities, limited recognition within work processes, and difficulties in interpersonal relationships within teams. Uncertainties regarding labor market insertion after residency were also highlighted ^28^^,^^29^. This context broadens debates on mental health and the career paths of graduates ^30^.

These challenges coexist with positive effects identified in residency programs that strengthen SUS services and care networks. Notable effects include expanded access, reduced avoidable hospitalizations, strengthened resolvability of primary health care, improved quality of care, and encouragement of collaborative and interprofessional practices. They also contribute to the reorganization of work processes, care innovation, strengthening of bonds with users, and integration between education and service. 

However, consolidating residencies as a structuring strategy within the SUS requires acknowledging the tensions surrounding their implementation, as well as the limits imposed by institutional models that are not always prepared to sustain the pedagogical assumptions and the ethical and political commitments of these programs. Consolidating residencies within the SUS requires expanding positions, valuing preceptorship, qualifying practice settings, and recognizing residents as active subjects in training.

## Data Availability

An online repository containing the electronic spreadsheets used for full-text selection and data extraction was created and is available at: https://drive.google.com/drive/folders/1OfOk5TILreYuu2D30OiY-hcRexT8XNIT?usp=sharing. Additionally, the data were made available through the Open Science Framework project (doi.org/10.17605/OSF.IO/JZH3X).
