# Supplementary material for: Effects of health residency programs on services and care networks of the Brazilian Unified Health System: rapid scoping review, Brasília, 2025
Source: Epidemiol Serv Saude. 2026 Feb 26;35:e20250464. doi: 10.1590/S2237-96222026v35e20250464.en (PMC13686303; doi:10.1590/S2237-96222026v35e20250464.en)
Supplement: Supplementary file 2 [file 2237-9622-ress-35-e20250464-Suplementar-tabela2.pdf]

Tabela Suplementar 2. Lista de estudos excluídos e razão da exclusão (n=48)

**Não apresenta efeitos das residências no sistema de saúde (n=30)**	
1.	Araújo HPA, Santos LCD, Domingos TDS, Alencar RA. Multiprofessional family health residency as a setting for education and interprofessional practices. Rev Lat Am Enfermagem. 2021;29:e3450.
2.	Arnemann CT, Kruse MHL, Gastaldo D, Jorge ACR, Silva AL, Margarites AGF. Preceptor's best practices in a multiprofessional residency: interface with interprofessionality. Interface (Botucatu). 2018;22:1635-46.
3.	Cahú RAG, Santos ACO, Pereira RC, Vieira CJL, Gomes SA. Stress and quality of life in multi-professional residency. Rev Bras Ter Cogn. 2014;10(2):76-83.
4.	Carvalho MAP, Gutiérrez AC. Fifteen years of Multiprofessional Residency in Family Health at Primary Health Care: contributions from Fiocruz. Cien Saude Colet. 2021;26(6):2013-22.
5.	Casanova IA, Batista NA, Moreno LR. A Educação Interprofissional e a prática compartilhada em programas de residência multiprofissional em Saúde. Interface (Botucatu). 2018;22(Suppl 1):1325-37.
6.	Castro RCL, Diercks MLMS, Corrêa FA, Gyrão NB, Pekelman R, Umpierre RN. O programa teórico da residência em Medicina de Família e Comunidade do Serviço de Saúde Comunitária do Grupo Hospitalar Conceição, Porto Alegre, RS. Rev Bras Med Fam Comunidade. 2014;9(33):375-83.
7.	Catalanotti JS, Popiel DK, Duwell MM, Price JH, Miles JC. Public health training in internal medicine residency programs: a national survey. Am J Prev Med. 2014;47(5 Suppl 3):S360-7.
8.	Damiance PRM, Tonete VLP, Daibem AML, Ferreira MDLDSM, Bastos JRDM. Formação para o SUS: uma análise sobre as concepções e práticas pedagógicas em saúde coletiva. Trab Educ Saúde. 2016;14(3):699-721.
9.	Demogalski JT, Alves FBT, Cabral LPA, Grden CRB, Bordin D, Fadel CB. Qualificação da residência multiprofissional em saúde: opiniões críticas de preceptores. Rev Pesqui (Univ Fed Estado Rio J Online). 2021;13:136-43.
10.	Fernandes MNS, Beck CL, Weiller TH, Viero V, Freitas PH, Prestes FC. Suffering and pleasure in the process of forming multidisciplinary health residents. Rev Gaúcha Enferm. 2015;36:90-7.
11.	Filho EJS, Sampaio J, Braga LAV. A avaliação de um programa de residência multiprofissional em Saúde da Família e a comunidade sob o olhar dos residentes. Tempus Actas Saúde Coletiva. 2016;10(4):129-49.
12.	Flor TBM, Miranda NM, Marinho CDSR, Pinheiro JMF, Sette-de-Souza PH, Noro LRA. Admission of alumni from Multiprofessional Residency Programs into the SUS. Rev Saude Publica. 2021;55:88.
13.	Flor TBM. Formação e inserção profissional de residentes multiprofissionais egressos de programas voltados à atenção básica. 2021;0:194.
14.	Freire JM, Infante A, Aguiar AC, Carbajo P. An analysis of the medical specialty training system in Spain. Hum Resour Health. 2015;13:42.
15.	Grunebaum A, Minkoff H, Blake D. Pregnancy among obstetricians: a comparison of births before, during, and after residency. Am J Obstet Gynecol. 1987;157(1):79-83.
16.	Inga-Berrospi F, Toro-Huamanchumo CJ, Sánchez LA, Torres-Vigo V, Taype-Rondán A. Características de la residencia médica en sedes docentes de Lima, Perú. Rev Cubana Educ Med Super. 2016;30(2).
17.	Izecksohn MMV, Teixeira JE, Stelet BP, Jantsch AG. Preceptorship in Family and Community Medicine: challenges and achievements in a Primary Health Care in progress. Cien Saude Colet. 2017;22:737-46.
18.	Lourenção LG. Work engagement among participants of residency and professional development programs in nursing. Rev Bras Enferm. 2018;71:1487-92.

19.	Miranda NMV, Leonello VM, Oliveira MA. Multiprofessional residency in health: a document analysis of political pedagogical projects. Rev Bras Enferm. 2015;68:586-93.
20.	Motta LB, Pacheco LC. Integrating medical and health multiprofessional residency programs: the experience in building an interprofessional curriculum for health professionals in Brazil. Educ Health (Abingdon). 2014;27(1):83-8.
21.	Mueller V. O papel das Residências em Saúde na qualificação e expansão da Atenção Primária: saberes e impressões na perspectiva do usuário [monografia]. 2018;0:99f.
22.	Oliveira AMFD, Moreira MRC, Xavier SPL, Machado MDFAS. Análise da integração ensino-serviço para a formação de residentes em medicina de família e comunidade. Rev Bras Educ Med. 2021;45(1):e003.
23.	Oliveira LFMM, Ramallo DA, Möller JVS, Leal AC, Ribeiro GA, Guimarães JAM. The role of the resident doctor in orthopedics and traumatology in a large hospital of the Unified Health System: what is the user's view? Rev Bras Ortop. 2020;56(4):438-45.
24.	Pasini VL, Pretto AMP, Sarria AM, Cardoso MFS. Perfil de egressos de residências multiprofissionais em saúde no Rio Grande do Sul. Rev Polis Psique. 2020;10(3):205-25.
25.	Ribeiro ER. The teaching and learning process of multiprofessional residents on patient safety and quality of health care: a systematic review. Medp Public Health Epidemiol. 2023;2(1).
26.	Ribeiro LG, Cyrino EG, Pazin-Filho A. Improving the Quality of Residency Programs in Family and Community Medicine. Rev Saude Publica. 2023;57:65.
27.	Santos IG, Batista NA, Devincenzi MU. Multiprofessional residency in family health: the conceptions of healthcare professionals regarding nutritionists' performance. Interface (Botucatu). 2015;19(53):349-60.
28.	Silva CT, Terra MG, Kruse MHL, Camponogara S, Xavier MS. Multi-professional residency as an intercessor for continuing education in health. Texto Contexto Enferm. 2016;25(1).
29.	Silva JC, Contim D, Ohl RI, Chavaglia SR, Amaral EM. Perception of the residents about their performance in the multidisciplinary residency program. Acta Paul Enferm. 2015;28(2):132-8.
30.	Silva YEM. Perfil e trajetória profissional dos egressos da residência multiprofissional em saúde coletiva [Trabalho de Conclusão de Curso]. Recife: Instituto Aggeu Magalhães, Fundação Oswaldo Cruz; 2021.
**Não é sistema de saúde semelhante ao do Brasil (n=6)**	
31.	Bieck AD, Biggs WS, Crosley PW, Kozakowski SM. Results of the 2012 National Resident Matching Program: family medicine. Fam Med. 2012;44(9):615-9.
32.	Biggs WS, Crosley PW, Kozakowski SM. Results of the 2013 National Resident Matching Program. Fam Med. 2013;45(9):647-51.
33.	Brown CM, Cronholm PF, Wright J, Warning WJ 2nd, Radosh L, Gabbay R. A collaborative approach to achieving NCQA PCMH recognition within family medicine residency practices. Fam Med. 2014;46(1):19-27.
34.	Diaz VA, Chessman A, Johnson AH, Brock CD, Gavin JK. Balint groups in family medicine residency programs: a follow-up study from 1990–2010. Fam Med. 2015;47(5):367-72.
35.	Hewitt DB, Chung JW, Ellis RJ, Cheung EO, Moskowitz JT, Hu YY. National evaluation of surgical resident grit and the association with wellness outcomes. JAMA Surg. 2021;156(9):856-63.
36.	Zeigler C, Jacobs ZG, Schwanke Khilji SU, Rice MK, Frink B, Carney PA. Increasing care for underserved communities through a global health residency training program. Ann Glob Health. 2024;90(1):70.
**Ensaio conceitual (n=4)**	

37.	Bernardo MDS, Fabrizzio GC, Souza ML, Santos TOD, Andrade SRD. Training and work process in Multiprofessional Residency in Health as innovative strategy. Rev Bras Enferm. 2020;73:e20190635.
38.	Fiorano AMM, Guarnieri AP. Residência multiprofissional em saúde: tem valido a pena? ABCS Health Sci. 2015;40(3):366-9.
39.	Fortuna CM, Mishima SM, Matumoto S, Pereira MJ, Ogata MN. The research and association between teaching and care in the consolidation of the Brazilian National Health System. Rev Esc Enferm USP. 2011;45:1696-700.
40.	Rossoni E. Residência na atenção básica à saúde em tempos líquidos. Physis. 2015;25(3):1011-31.
**Duplicada manual (n=3)**	
41.	Jantsch AG, Burström B, Nilsson GH, Leon AP. The impact of residency training in family medicine on hospital admissions due to Ambulatory-care Sensitive Conditions in Rio de Janeiro. PLOS Glob Public Health. 2023;3(10):e0000547.
42.	Pinto TR, Cyrino EG. Os Programas de Residência Multiprofissional em Saúde na conformação das redes prioritárias de atenção. Interface (Botucatu). 2022;26:e200770.
43.	Santos JS, Santos Neto PMD. Training for the Brazilian National Health System (SUS) in health residency programs: an analysis of policy effects in a state context. Interface (Botucatu). 2024;28:e230587.
**Estudo indisponível (n=2)**	
44.	Fiszbeyn C. A residência médica como formadora de recursos humanos para o Sistema de Saúde no Estado de São Paulo: uma visão crítica [tese]. São Paulo: Universidade de São Paulo; 2000.
45.	Sampaio SAP, Gallucci LFL, Conceição SB, Sant'Anna VM. O programa de residência médica do Governo do Estado de São Paulo: análise histórica e tendências de desenvolvimento. Cad FUNDAP. 1997;21:216-27.
**Relato de experiência/estudo de caso (n=2)**	
47.	Nascimento DDG. A residência multiprofissional em saúde da família como estratégia de formação da força de trabalho para o SUS [dissertação]. São Paulo: Universidade de São Paulo, Escola de Enfermagem; 2008.
48.	Oyama DHM, Campos EC. A primeira residência brasileira de medicina de família dentro de uma instituição privada: a experiência das residentes. Mundo Saúde. 2010;34(3):384-8.
