# Supplementary material for: Effects of health residency programs on services and care networks of the Brazilian Unified Health System: rapid scoping review, Brasília, 2025
Source: Epidemiol Serv Saude. 2026 Feb 26;35:e20250464. doi: 10.1590/S2237-96222026v35e20250464.en (PMC13686303; doi:10.1590/S2237-96222026v35e20250464.en)
Supplement: Supplementary file 3 [file 2237-9622-ress-35-e20250464-Suplementar-tabela3.pdf]

Material Suplementar

Tabela Suplementar 3. Avaliação da qualidade, resolutividade e práticas em saúde nos estudos incluídos (n=12)

Autor, ano de publicação	Resultado dos programas	
	Qualidade e resolutividade nos serviços	Integração ensino-serviço, educação permanente, boas práticas em saúde, prática interprofissional
**Estudos qualitativos**		
Barreto, 2019	**Resolutividade:** A ampliação da cobertura de médicos de família nas unidades básicas de saúde deu maior acesso e resolutividade. Aumento do número de municípios oferecendo residência médica em saúde da família, passando de 2 municípios em 2010 para 24 em 2017. A expansão da residência resultou na qualificação da atenção básica e no aumento da participação de municípios no processo de formação médica.	**Integração ensino-serviço**: Residências médicas passaram a atuar como dispositivos estruturantes da formação profissional e da fixação de médicos no SUS[a].
Fernandes, 2015	**Qualidade do serviço:** Observou-se persistentemente nos discursos a melhoria da qualidade na assistência aos pacientes após a implantação do Programa de Residência em Anestesiologia, com indução da prática reflexiva pelos preceptores, motivada pelas constantes indagações e pelos constantes questionamentos dos residentes, como também mediante a abordagem mais completa e integrada dos pacientes, em detrimento da abordagem puramente tecnicista e intraoperatória.	**Educação permanente:** Foi enfatizado que a presença do residente motivou os profissionais anestesiologistas da referida instituição na busca de atualização e despertou a necessidade de se envolver com processos de educação continuada.<br><br>**Boas práticas em saúde:** Prática interprofissional – o residente atua como um elo interdisciplinar nos cuidados perioperatórios. Nessa categoria foi evidenciada recorrentemente a melhoria da interlocução anestesiologista-cirurgião acerca do planejamento e da escolha da técnica anestésica baseados no procedimento cirúrgico proposto.

Landim, 2012	**Qualidade do serviço:** A vivência hospitalar no contexto da residência multiprofissional em saúde da família foi importante diante da possibilidade de aproximar as residentes da sua esfera de trabalho na atenção primária à saúde. Propostas como a residência multiprofissional são potencialmente geradoras de mudanças para os profissionais já atuantes, considerando a possibilidade de os residentes motivá-los para a reflexão de sua prática.	**Educação permanente:** Foi possível refletir sobre a vivência hospitalar e a atenção primária à saúde, compreendendo que as residentes mobilizam e influenciam os membros da equipe a reverem e adquirirem novos conhecimentos, fortalecendo um espaço para educação permanente.<br><br>**Integração ensino-serviço:** A vivência hospitalar auxilia no processo de ensino-aprendizagem, favorecendo a incorporação de novos conhecimentos, através da aproximação da realidade. Assim, o programa de residência, ao diversificar cenários de ensino-aprendizagem, tem contribuído para o desenvolvimento e/ou aprimoramento de competências.
Machado, 2018	Não relatado.	**Integração ensino-serviço**: As ações desenvolvidas no campo de atuação devem ser condizentes com os contextos de ensino e trabalho, o que pressupõe iniciativas adequadas à realidade contextual, possibilitando o desenvolvimento das competências desejadas e promovendo constante atualização para o bom desempenho profissional.
Pinheiro, 2023	Não relatado.	**Boas práticas em saúde:** A residência fomenta debates sobre a relação entre enfermagem e SUS, abordando temas como participação popular, formação sanitarista e financiamento adequado.<br><br>**Integração ensino-serviço:** A residência promove transformações no serviço e no território, estimulando debates sobre o fortalecimento e a defesa do SUS. A formação dos residentes envolve atividades coletivas, como promoção e educação em saúde, metodologias ativas, estudos teóricos, pesquisas e intervenções no território.

Pinto, 2022	**Qualidade do serviço:** Mesmo sendo programas direcionados à atenção primária à saúde, têm possibilitado aos seus residentes estabelecer novas pontes e conexões entre os diferentes âmbitos de atenção no SUS, favorecendo a verdadeira rede poliárquica com múltiplas portas e conexões.	**Integração ensino-serviço:** Atribuiu-se aos programas de residência multiprofissional a perspectiva de pós-graduação que oportuniza a apropriação teórica e o exercício da prática.<br><br>**Prática interprofissional:** Destacou-se o papel dos programas de residência multiprofissional em fomentar o crescimento e a ampliação do potencial de atuação profissional pelo contato e pela interação com outras categorias que, ao somar esforços, se veem em condições de alavancar as fronteiras do conhecimento e das possibilidades de atuação.
Santos, 2024	**Qualidade do serviço:** A política de residências em áreas profissionais da saúde em Pernambuco tem possibilitado, de forma mais imediata, a ampliação do número de profissionais e de especialidades; e o “reconhecimento direto com o usuário”. A maior disponibilidade de profissionais e a diversificação das profissões resultam na percepção de ampliação do acesso dos usuários às ações e aos serviços no SUS e na melhoria da qualidade da atenção, como verificado também por outros estudos.	**Redução de desigualdades regionais:** A política de residências em áreas profissionais da saúde em Pernambuco tem como um dos efeitos ordenar a formação de profissionais para uma rede de saúde regionalizada, contribuindo para reduzir as desigualdades na oferta de formação e promover transformações nos serviços.<br><br>**Boas práticas em saúde:** Formação crítica e reflexiva – a residência em saúde se destaca por integrar teoria e prática, diferenciando-se de outros processos educacionais, incluindo a graduação. Espera-se que os egressos desenvolvam pensamento crítico, competência técnica e compromisso com a melhoria do SUS. Como pós-graduação voltada para a prática, a residência complementa a formação acadêmica e prepara profissionais para uma atuação mais abrangente.<br><br>**Aprimoramento da assistência:** É possível identificar algumas contradições quanto aos efeitos esperados do Programa de Residência em Área Profissional da Saúde: se, por um lado, contribui para qualificar a atuação dos profissionais no SUS, por outro, colabora para emergir a dificuldade de absorção dos egressos dos programas de residência por carência de concursos e de salários; e por condições de trabalho inadequadas.

Souza, 2022	**Resolutividade:** A inserção dos residentes nas unidades básicas de saúde tem contribuído significativamente para a ampliação da resolutividade. Gestores relatam melhorias estruturais e assistenciais nos serviços; preceptores apontam avanços na vigilância, na articulação intersetorial e nas ações coletivas; residentes reconhecem seu papel como agentes transformadores; e usuários destacam a ampliação do acesso, da escuta e da capacidade de resposta das equipes de saúde.	**Integração ensino-serviço:**<br>A residência foi apontada como um marco importante no desenvolvimento profissional dos residentes, impulsionando sua inserção em atividades de pesquisa, elaboração de projetos e participação em eventos científicos, o que fortaleceu sua trajetória acadêmica e técnica.
**Estudos quantitativos**		
Costa, 2021	**Qualidade do serviço:** Os baixos escores de qualidade foram atribuídos a dificuldades de acesso, baixa integralidade e centralidade do cuidado, fragilidade na comunicação entre níveis de atenção, pouca resolutividade das unidades e limitada participação comunitária nas ações e no controle social.	**Aprimoramento da assistência:** Observou-se a influência positiva no escore geral de percepção dos usuários sobre a qualidade da atenção os fatores: estratégia saúde da família completas, maior número de agentes comunitários de saúde e presença de residência de medicina de família e comunidade.<br><br>Ter residência de medicina de família e comunidade teve impacto positivo no escore geral (aumento de 0,635; p 0,005; IC95%[b] 0,2; 1,1) e no escore essencial (aumento de 0,527; p 0,018; IC95% 0,1; 1,0).
Christofoletti, 2015	**Resolutividade:** A assistência multiprofissional ao paciente e seus familiares mostrou-se positiva, com 65,2% (n=30) dos pacientes assistidos recebendo alta hospitalar.	**Integração ensino-serviço:** A diversidade das condições apresentadas pelos usuários assistidos pela equipe da residência multiprofissional em saúde durante o período permitiu aos residentes uma experiência profissional com pacientes de diferentes especificidades clínicas. Isso contribuiu para a ampliação da visão sobre o processo terapêutico e, principalmente, para o estímulo à prática do trabalho multiprofissional integrado, baseado na clínica ampliada e nas necessidades de saúde de cada usuário.

Jantsch, 2023	**Resolutividade:** Com dois anos de treinamento em medicina de família, os médicos de família que trabalham na atenção primária podem reduzir o risco de seus pacientes serem hospitalizados para todas as 14 condições de saúde da lista brasileira de Condições Sensíveis de Atendimento Ambulatorial (ICSAP-Brasil), exceto para infecções de ouvido, nariz e garganta.	Não relatado.
**Estudos mistos (qualitativos e quantitativos)**		
Santos, 2023	**Resolutividade:** A ampliação das residências, especialmente as multiprofissionais, contribuiu para a organização da atenção primária e hospitalar, favorecendo a continuidade do cuidado. Além disso, o fortalecimento dos espaços de governança das residências, como fóruns estaduais e comissões de residência, permitiu maior articulação entre serviços e gestores, e a qualificação dos profissionais residentes.	**Integração ensino-serviço:** A residência fortaleceu a formação em serviço e a prática interprofissional, promovendo o ensino baseado em evidências e focado na realidade do SUS.

Notas: [a]SUS: Sistema Único de Saúde; [b]IC95%: intervalo de confiança de 95%.
