# Supplementary material for: Effects of health residency programs on services and care networks of the Brazilian Unified Health System: rapid scoping review, Brasília, 2025
Source: Epidemiol Serv Saude. 2026 Feb 26;35:e20250464. doi: 10.1590/S2237-96222026v35e20250464.en (PMC13686303; doi:10.1590/S2237-96222026v35e20250464.en)
Supplement: Supplementary file 4 [file 2237-9622-ress-35-e20250464-Supplementary-table1.pdf]

Tabela Suplementar 1. Estratégia de busca utilizada em cada fonte (n=12)

Fonte	Estratégia de busca	Total de referências recuperadas
**PubMed**	("Internship and Residency"[Mesh] OR residency OR residencies OR "health residency" OR "medical residency" OR "multiprofessional residency" OR "residency programs" OR "medical training") AND ("Unified Health System" OR "Sistema Unico de Saude" OR "SUS" OR "National Health System" OR "Public Health System")	1.008
**Web of Science**	("Internship and Residency" OR residency OR residencies OR "health residency" OR "medical residency" OR "multiprofessional residency" OR "residency programs" OR "medical training") AND ("Unified Health System" OR "Sistema Unico de Saude" OR "SUS" OR "National Health System" OR "Public Health System")	163
**BVS**	("Internship and Residency" OR "Internato e Residência" OR "Internado y Residencia" OR residency OR residencies OR residência OR residências OR "health residency" OR "medical residency" OR "multiprofessional residency" OR "residency programs" OR "medical training" OR "Residência Hospitalar" OR "Residência Multidisciplinar" OR "Residência Médica" OR "Residência em Saúde") AND ("Unified Health System" OR "Sistema Unico de Saude" OR "SUS" OR "National Health System" OR "Public Health System" OR "sistema de saúde nacional" OR "sistema de saúde público") AND (impact OR impacto OR quality OR qualidade OR strength* OR fortalecimento OR satisfaction OR satisfação OR accessibility OR acessibilidade OR resolution OR resolutividade OR access OR acesso OR "health promotion" OR "promoção de saúde" OR "health education" OR "care coordination" OR "health care") AND db:("LILACS") AND instance:"lilacsplus"	1.292

**Google Scholar**	("Internship and Residency" OR residency OR residencies OR "health residency" OR "medical residency" OR "multiprofessional residency" OR "residency programs" OR "medical training") AND ("Unified Health System" OR "Sistema Único de Saúde" OR "SUS" OR "National Health System" OR "Public Health System") AND (impact OR quality OR strength* OR satisfaction OR accessibility OR resolution OR access OR "health promotion" OR "health education" OR "care coordination" OR "health care")	100 primeiros resultados
**Total= 2.563**		
