# Supplementary material for: Effects of health residency programs on services and care networks of the Brazilian Unified Health System: rapid scoping review, Brasília, 2025
Source: Epidemiol Serv Saude. 2026 Feb 26;35:e20250464. doi: 10.1590/S2237-96222026v35e20250464.en (PMC13686303; doi:10.1590/S2237-96222026v35e20250464.en)
Supplement: Translation

# Translation: Efeitos dos programas de residências em saúde nos serviços e nas redes de atenção do Sistema Único de Saúde: revisão de escopo rápida, Brasília, 2025

**DOI:** 10.1590/S2237-96222026v35e20250464.pt

**Authors:** Pedro Emanuel do Nascimento Fernandes, Ana Gabriela Costa Normando, Keitty Regina Cordeiro de Andrade, Fabianne de Castro Teixeira, Priscilla Azevedo Souza, Paloma Ribeiro Pires Simas, Denise Fernandes Leite, Fernando Canto Michelotti, Michele Sacramento dos Santos, Fernanda Madeira de Ley Botelho Cunha, Marta Roberta Santana Coelho, Fábio Henrique Cavalcanti de Oliveira

**Affiliations:** 1 Ministério da Saúde, Departamento de Ciência e Tecnologia, Brasília, DF, Brasil Ministério da Saúde Departamento de Ciência e Tecnologia Brasília DF Brasil; 2 Ministério da Saúde, Departamento de Gestão da Educação na Saúde, Brasília, DF, Brasil Ministério da Saúde Departamento de Gestão da Educação na Saúde Brasília DF Brasil

**Keywords:** Internato e Residência, Sistemas de Saúde, Serviços de Saúde, Revisão de Escopo, Brasil.

## Abstract

**Objetivo:**

Investigar os efeitos dos programas de residência, para os serviços e as redes de atenção do Sistema Único de Saúde (SUS).

**Métodos:**

Trata-se de revisão de escopo rápida conduzida segundo o Instituto Joanna Briggs e o checklist Preferred Reporting Items for Systematic Reviews and Meta-Analysis extension for Scoping Reviews. As buscas foram realizadas em janeiro de 2025, sem restrição temporal, nas bases PubMed, Web of Science e Biblioteca Virtual em Saúde, além da literatura cinzenta via Google Scholar. A seleção ocorreu na ferramenta Rayyan, incluindo artigos completos em inglês, português e espanhol. Os dados extraídos foram analisados por meio de abordagem narrativa.

**Resultados:**

De 2.563 estudos identificados, 12 artigos foram incluídos, com publicações entre 2012 e 2024. Os estudos contemplaram programas de residência em diferentes regiões do Brasil, com destaque para o Nordeste, incluindo residência multiprofissional, médica e em medicina de família e comunidade. A síntese dos resultados foi organizada em cinco eixos: (i) resolutividade dos serviços; (ii) qualidade da atenção; (iii) integração entre ensino e serviço; (iv) aprimoramento da assistência; e (v) disseminação de boas práticas. Evidenciaram-se impactos positivos das residências na ampliação do acesso, qualificação clínica e indução de práticas interprofissionais e colaborativas. A integração ensino-serviço fortaleceu a resolutividade ao reorganizar processos de trabalho, estimular reflexão crítica e alinhar a atuação aos princípios do SUS. A presença de residentes impulsionou inovações, fortaleceu vínculos com usuários e promoveu transformações assistenciais.

**Conclusão:**

As residências contribuem para qualificar a atenção no SUS, ao promoverem acesso, resolutividade, qualidade, práticas colaborativas e inovação, o que evidencia seu papel estruturante nas redes de atenção.

**Aspectos éticos**

Esta pesquisa utilizou bancos de dados de domínio público e anonimizados.

## Introdução 

A formação de profissionais qualificados constitui um dos pilares fundamentais para a consolidação do Sistema Único de Saúde (SUS), na medida em que estrutura uma força de trabalho capaz de responder às necessidades de saúde da população com qualidade, integralidade e equidade^1^. Nesse contexto, os programas de residência em saúde (médicos, multiprofissionais e uniprofissionais) têm se destacado como estratégias potentes de educação permanente e qualificação profissional, ao promoverem a inserção de profissionais em formação diretamente nos serviços do SUS ^2^^,^^3^.

Instituídas como modalidades de pós-graduação lato sensu, as residências na área profissional da saúde têm como característica central o ensino em serviço, fundamentado nos princípios do SUS e voltado para a atuação junto a usuários, famílias e comunidades em seus contextos reais de vida ^4^. Essa imersão prática proporciona a formação crítica e situada, que articula o conhecimento técnico-científico com a realidade dos territórios. Isso possibilita que os profissionais desenvolvam competências clínicas, gerenciais, éticas e político-institucionais em diálogo constante com os desafios dos serviços de saúde ^5^.

A presença de residentes nos serviços públicos tem o potencial de estimular transformações organizacionais, promovendo a adoção de práticas baseadas em evidências, a qualificação dos processos de cuidado e a ampliação da capacidade de resposta das equipes ^6^. Além disso, pode favorecer a renovação da força de trabalho, por meio da atualização contínua de condutas clínicas e da incorporação de diretrizes técnico-políticas recentes, o que contribui para a consolidação da cultura de educação permanente ^6^^,^^7^.

As residências em saúde se projetam como estratégia formativa que pode auxiliar na ampliação de vagas em áreas prioritárias, na valorização da atuação docente-assistencial, na promoção da cidadania e na articulação interprofissional nos serviços ^6^. Essas residências visam qualificar a formação com foco em competências alinhadas às necessidades dos territórios, promover o enfrentamento a discriminações estruturais, fomentar o cuidado centrado no usuário e fortalecer a integração entre ensino, serviço e comunidade ^8^.

Ao mesmo tempo, os programas de residência também têm sido alvo de críticas relacionadas às fragilidades estruturais e organizacionais dos cenários de prática, muitas vezes marcados por carências de infraestrutura, escassez de recursos materiais e humanos, sobrecarga das equipes e ausência de planejamento pedagógico adequado ^2^. Essas questões impactam, de maneira direta, a preceptoria e a tutoria, especialmente diante da precarização da função do preceptor, o que compromete o acompanhamento efetivo dos residentes ^9^.

Nos últimos anos, também ganhou destaque a preocupação com as condições de saúde mental dos residentes, frequentemente expostos a jornadas extenuantes, situações de violência institucional e assédio, ausência de apoio psicossocial e vínculos de trabalho precários ^10^. Considerando-se todo o contexto, o objetivo deste estudo foi investigar os efeitos dos programas de residência para os serviços e as redes de atenção do SUS.

## Métodos 

Delineamento

Trata-se de revisão de escopo rápida, que utiliza atalhos metodológicos na condução de algumas etapas de elaboração de uma revisão escopo tradicional. Tais atalhos possibilitam a diminuição do tempo de elaboração. 

Esta revisão de escopo rápida foi construída de acordo com a metodologia desenvolvida pelo Reviewer’s Manual for Scoping Reviews do Instituto Joanna Briggs ^11^. Esta revisão foi relatada de acordo com as diretrizes Preferred Reporting Items for Systematic Reviews and Meta-Analysis extension for Scoping Reviews ^12^.

A pergunta de pesquisa que orientou esta revisão foi: ‘Quais são os efeitos dos programas de residências em saúde (médicas e multiprofissionais/uniprofissionais) na qualidade e/ou resolutividade dos serviços e redes de atenção do Sistema Único de Saúde (SUS)?‘. Essa pergunta foi elaborada com base no acrônimo PCC (população, conceito e contexto), conforme a seguir. 

População: programas de residência em saúde. 

Conceito: efeitos na qualidade e resolutividade dos serviços e das redes de atenção. 

Contexto: Sistema Único de Saúde brasileiro. 

Critérios de elegibilidade

Foram incluídos: (i) estudos sobre efeitos das residências na qualidade, na resolutividade e nos resultados nos serviços do sistema público de saúde brasileiro; (ii) os delineamentos metodológicos como estudos observacionais (transversais, de coorte ou de caso-controle), estudos de avaliação e/ou implementação de programas ou políticas, estudos exploratório-descritivos com abordagem qualitativa, quantitativa ou mista, bem como revisões sistemáticas, integrativas ou de escopo; (iii) documentos institucionais que apresentassem dados sistematizados sobre os efeitos das residências nos serviços de saúde; e (iv) publicações nos idiomas português, inglês ou espanhol, independentemente do ano de publicação. Foram excluídos: (i) estudos que não abordassem programas de residência em saúde vinculados a sistemas públicos de saúde; (ii) revisões narrativas, análises documentais, documentos institucionais sem dados empíricos, ensaios conceituais ou relatos de experiência; (iii) estudos que não apresentassem dados relativos à qualidade e/ou à resolutividade dos serviços ou das redes de atenção; (iv) publicações disponibilizadas apenas como resumos de congressos, editoriais, comentários ou opiniões de especialistas; e (v) estudos não disponíveis na íntegra.

Fontes de informação 

As buscas foram realizadas nas bases de dados Medline (via PubMed), Web of Science e Biblioteca Virtual em Saúde. Além disso, foram realizadas buscas no Google Scholar, abrangendo tanto artigos científicos quanto literatura cinzenta (teses e dissertações). 

Optou-se por não realizar a busca manual nas referências de publicações relevantes para identificar estudos adicionais potencialmente elegíveis, considerando o caráter de revisão de escopo rápida deste estudo. Não foram aplicadas restrições quanto ao idioma ou ao status de publicação.

Estratégia de busca 

Uma estratégia-piloto foi desenvolvida para a base de dados Medline (via PubMed) e adaptada para as demais bases de dados e para o Google Scholar. Apresentou-se a estratégia final para cada base de dados (Tabela Suplementar 1). Os resultados da busca foram importados para o software Rayyan ^13^ com a finalidade de remover as duplicações e prosseguir com as etapas de revisão. As buscas em todas as bases de dados foram realizadas em 15 de janeiro de 2025.

Processo de seleção 

Três pesquisadores (PENF, AGCN e KRCA) realizaram a seleção dos estudos de forma independente, não pareada e não cega, com base nos títulos e resumos por meio da plataforma Rayyan ^13^. No Google Scholar, foram consideradas as 100 primeiras publicações identificadas para essa etapa inicial ordenadas por relevância. Em seguida, os textos completos dos estudos elegíveis foram analisados em uma planilha eletrônica previamente testada e calibrada. Eventuais discrepâncias ou dúvidas foram resolvidas por consenso em reunião conjunta entre os três pesquisadores.

Processo de extração de dados e itens

Três pesquisadores (PENF, AGCN e KRCA) realizaram a extração dos dados de forma independente, utilizando uma planilha eletrônica padronizada e previamente testada. O processo foi calibrado em reunião inicial.

Itens de dados

Dados sobre as características gerais dos programas de residência e dos serviços de saúde foram extraídos, conforme descrito a seguir.

Características gerais: autor, ano, local de realização, instituição, delineamento e período do estudo. Também foram considerados o perfil dos participantes (usuários, residentes, preceptores, tutores, coordenadores, gestores e trabalhadores do SUS ou de outros sistemas públicos), o tipo de serviço de saúde e sua relação com o sistema.

Características dos programas de residência: modalidade da residência (médica, multiprofissional ou uniprofissional), categoria profissional, especialidade, área de atuação, região geográfica (urbana, rural ou prioritária), duração e carga horária.

Características referentes aos serviços de saúde: indicadores de qualidade (satisfação dos usuários, acessibilidade, continuidade e coordenação do cuidado) e de resolutividade (redução de filas, impacto em indicadores, solução de problemas). Foram também analisados os resultados relacionados à integração ensino-serviço, ao estímulo à produção científica e boas práticas, à qualificação profissional, à valorização da preceptoria e à redução das desigualdades regionais.

Avaliação crítica de fontes individuais de evidências

Neste estudo, não foi realizada a análise do risco de viés dos estudos incluídos.

Síntese dos dados 

Os dados extraídos foram sintetizados com base nos resultados dos estudos e organizados em cinco eixos temáticos, definidos a partir da recorrência e relevância das informações: (i) resolutividade dos serviços; (ii) qualidade da atenção em saúde; (iii) integração entre ensino e serviço; (iv) aprimoramento da assistência; e (v) disseminação de boas práticas em saúde. Os achados foram apresentados de forma narrativa, e nenhuma meta-análise foi realizada devido à natureza qualitativa dos dados. A elaboração e a discussão dos resultados foram conduzidas por todos os autores (PENF, AGCN e KRCA).

**Tabela 1:** Características dos estudos incluídos (n=12). Brasília, 2025

Autor, ano de publicação	Caracterização dos estudos		Características da população	Caracterização dos programas de residência	
	Delineamento	Local de realização	Participantes	Modalidade do programa de residência	Nível de atenção
Estudos qualitativos					
Barreto, 2019	Sistematização de experiência	Paraíba, Brasil	Professores e pesquisadores	Residência médica em medicina de família e comunidade	Atenção primária à saúde
Fernandes, 2015	Representação Social	Ceará, Brasil	Profissionais de saúde	Residência médica em anestesiologia	Atenção terciária à saúde
Landim, 2012	Referencial teórico-metodológico da fenomenologia	São Paulo, Brasil	Residentes enfermeiras	Programa de residência multiprofissional em saúde da família	Atenção primária à saúde
Machado, 2018	Descritivo exploratório	Ceará, Brasil	Residentes de enfermagem, psicologia, serviço social e educação física	Residência integrada com ênfase em saúde mental coletiva, saúde da família e comunidade e saúde coletiva	Atenção primária à saúde
Pinheiro, 2023	Descritivo exploratório	Ceará, Brasil	Enfermeiros residentes	Residência multiprofissional de saúde da família e comunidade	Atenção primária à saúde
Pinto, 2022	Descritivo exploratório	Rio Grande do Norte e São Paulo, Brasil	Coordenadores e residentes	Programas de residência multiprofissional em saúde	Atenção primária à saúde
Santos, 2024	Pesquisa documental e entrevistas	Pernambuco, Brasil	Gestores, preceptores, residentes e controle social	Política de residência em área profissional da saúde de Pernambuco	Todos os níveis de atenção
Souza, 2022	Estudo de caso com abordagem qualitativa	Pernambuco, Brasil	Gestores, preceptores, residentes e usuários em comunidades quilombolas	Residência multiprofissional de saúde	Atenção primária à saúde
Estudos quantitativos					
Christofoletti, 2015	Transversal	Mato Grosso do Sul, Brasil	Usuários	Residência multiprofissional em saúde	Atenção terciária à saúde
Costa, 2021	Transversal	Ceará, Brasil	Usuários	Residência de medicina de família e comunidade	Atenção primária à saúde
Jantsch, 2023	Análise longitudinal retrospectiva	Rio de Janeiro, Brasil	Usuários	Residência médica em medicina de família e comunidade	Atenção primária à saúde
Estudos mistos (qualitativos e quantitativos)					
Santos, 2023	Estudo de caso de abordagem mista	Pernambuco, Brasil	Atores de governança da residência em saúde	Residências uniprofissionais e multiprofissionais em saúde	Todos os níveis de atenção

## Resultados 

As buscas eletrônicas estruturadas resultaram na identificação de 2.563 estudos. Após a remoção de duplicatas, 2.423 estudos seguiram para a triagem por títulos e resumos. Destes, 60 estudos foram selecionados para leitura na íntegra, com base nos critérios de elegibilidade previamente estabelecidos. Ao final dessa etapa, 12 estudos atenderam aos critérios e foram incluídos na revisão ^14^^-^^25^. Os 48 estudos excluídos, juntamente com os motivos de exclusão, estão detalhados na Tabela Suplementar 2. O processo de seleção dos estudos está descrito na Figura 1. As teses e dissertações identificadas não atenderam aos critérios de elegibilidade e foram excluídas tanto na etapa de triagem inicial quanto na avaliação do texto completo.

Foram incluídos 12 estudos ^14^^-^^25^, sendo 8 de abordagem qualitativa ^14^^,^^17^^,^^19^^-^^22^^,^^24^^,^^25^, 3 de abordagem quantitativa ^15^^,^^16^^,^^18^ e 1 com métodos mistos (qualitativo e quantitativo) ^23^. As publicações dataram de 2012 a 2024 e contemplaram programas de residência implementados em diferentes regiões do Brasil. A região Nordeste foi a mais representada, com destaque para o Ceará, que concentrou 4 estudos ^16^^,^^17^^,^^20^^,^^21^, seguido de Pernambuco, com 3 ^23^^-^^25^. 

**Figura 1:** Processo de seleção e inclusão dos estudos na revisão de escopo. Brasília, 2025

Nos estudos identificados, todos os programas de residência ocorreram em instituições públicas. A maioria investigou programas de residência multiprofissional em saúde, envolvendo profissionais das áreas de enfermagem, psicologia, fisioterapia, fonoaudiologia, odontologia, nutrição, farmácia, educação física e serviço social ^15^^,^^19^^-^^25^. Também foram incluídos estudos sobre residência médica ^17^, com destaque para aqueles voltados à medicina de família e comunidade ^14^^,^^16^^,^^18^. 

Em termos de inserção nos serviços, a maior parte dos programas avaliados estava vinculada à atenção primária à saúde ^14^^,^^16^^,^^18^^-^^22^^,^^25^. Dois estudos investigaram programas situados em serviços de atenção terciária ^15^^,^^17^, e 2 abarcaram programas presentes em diferentes níveis de atenção ^23^^,^^24^. Apresentou-se o detalhamento das características dos estudos como local de realização, programa de residência e nível de atenção (Tabela 1).

A síntese dos resultados foi organizada com base nos temas mais recorrentes identificados nos estudos primários. O eixo da resolutividade dos serviços foi abordado em 6 estudos ^2^^,^^15^^,^^16^^,^^18^^,^^23^^,^^25^; a qualidade da atenção em saúde, em 5 ^16^^,^^17^^,^^19^^,^^22^^,^^24^; a integração entre ensino e serviço, em 8 ^2^^,^^15^^,^^19^^-^^23^^,^^25^; o aprimoramento da assistência, em 2 ^16^^,^^24^; e a disseminação de boas práticas em saúde, em 4 ^17^^,^^21^^,^^22^^,^^24^. A partir dessa frequência e relevância temática, os achados foram categorizados em 5 eixos analíticos que estruturaram a apresentação dos resultados: (i) resolutividade dos serviços; (ii) qualidade da atenção em saúde; (iii) integração entre ensino e serviço; (iv) aprimoramento da assistência; e (v) disseminação de boas práticas em saúde. O detalhamento das contribuições específicas de cada estudo incluído, por eixo temático, está disponível na Tabela Suplementar 3.

Os estudos analisados mostraram que os programas de residência em saúde impactam positivamente a resolutividade dos serviços do SUS ^25^. Destacaram-se a ampliação do acesso, a redução de internações evitáveis e a maior integração entre os níveis de atenção ^15^^,^^18^. Esses efeitos qualificaram o cuidado e fortaleceram as redes de atenção à saúde ^14^. A expansão da residência médica em medicina de família e comunidade foi apontada como elemento estruturante para o aumento da resolutividade na atenção primária à saúde ^25^. A presença ampliada de médicos de família nas unidades básicas de saúde elevou a cobertura assistencial, a capacidade de resolução de problemas e a participação dos municípios na formação médica e na gestão do cuidado ^14^.

Estudos quantitativos reforçaram esse impacto ao mostrar que médicos formados em residência médica em medicina de família e comunidade estão associados à redução de internações por condições sensíveis à atenção primária. Pessoas acompanhadas por esses profissionais apresentaram menor risco de hospitalização por doenças crônicas como hipertensão, diabetes, cardiovasculares e respiratórias, o que evidencia a potência da atenção primária à saúde na prevenção de agravos e hospitalizações ^15^^,^^18^.

Residentes multiprofissionais nas unidades básicas de saúde têm fortalecido a resolutividade local por meio da busca ativa, do fortalecimento de vínculos e da articulação entre profissionais e serviços da rede. A percepção de gestores, preceptores e usuários foi amplamente favorável, com destaque para melhorias nos serviços, maior acessibilidade e menor necessidade de encaminhamentos ^24^^,^^25^.

A presença de residentes nos serviços de saúde tem sido fator relevante para a qualificação da atenção, ao promover práticas reflexivas, integração interdisciplinar e ampliação do acesso ^17^^,^^22^. A atuação dos residentes estimula a troca de saberes, a reorganização dos processos de trabalho e o fortalecimento das redes de atenção à saúde ^24^. 

Na atenção terciária, a residência médica em anestesiologia contribuiu para mudanças concretas na prática assistencial, como a adoção de visitas pré-anestésicas, anteriormente inexistentes. Isso qualifica o cuidado perioperatório e fortalece a integração entre diferentes categorias profissionais. A presença dos residentes induziu reflexões críticas entre os preceptores e melhorias na segurança do usuário ^17^.

Nos programas de residência multiprofissional em saúde, relatos de residentes destacaram a capacidade desses programas de integrar diferentes serviços e fortalecer a formação em saúde no SUS, com impactos diretos sobre a qualificação das equipes e a efetivação das redes de atenção à saúde. Em particular, experiências hospitalares vivenciadas por enfermeiras durante a residência foram posteriormente aplicadas na atenção primária à saúde, o que influenciou positivamente a prática das equipes e promoveu espaços de educação permanente ^22^.

A avaliação da política estadual de residência em Pernambuco também evidenciou ganhos imediatos na qualidade da atenção, especialmente pelo aumento do número de profissionais especializados nos serviços. Isso resultou em maior reconhecimento por parte dos usuários e ampliação do acesso às ações do SUS ^23^^,^^24^.

A integração entre ensino e serviço se apresentou como componente central dos programas de residência em saúde, ao favorecer a articulação entre teoria e prática, fortalecer a formação profissional e qualificar os serviços no SUS ^25^. A inserção dos residentes nos cenários de cuidado contribui para a reorganização dos processos assistenciais, estimula a reflexão crítica e potencializa a educação permanente das equipes.

A residência proporciona experiências formativas que complementam lacunas deixadas pela graduação, ao aproximar os residentes da realidade dos serviços e das diretrizes do SUS ^14^. A diversidade de cenários e práticas, incluindo a vivência hospitalar e na atenção primária à saúde, favorece o desenvolvimento de competências técnicas, éticas e relacionais, em uma perspectiva contínua de aprendizagem ^21^.

A presença dos residentes também tem impulsionado inovações nos serviços, ao estimular práticas colaborativas, promover a construção coletiva do conhecimento e fortalecer a interação entre profissionais, gestores e instituições formadoras ^22^. A atuação conjunta tem sido reconhecida como fator de transformação da cultura institucional e da qualidade do cuidado.

Contudo, também foram evidenciados desafios importantes, como a fragilidade na articulação entre instituições de ensino e serviços de saúde, a ausência de apoio pedagógico sistemático e a precarização das funções de tutoria e preceptoria ^23^. Houve críticas quanto à falta de conexão entre os conteúdos curriculares e a realidade vivida pelos residentes nos serviços ^20^. 

A inserção de residentes nos serviços de saúde tem contribuído para o aprimoramento da assistência, ao aproximar a formação profissional da realidade dos territórios e das práticas do SUS ^16^. Essa imersão qualifica a clínica, amplia a oferta de serviços e fortalece a tomada de decisão das equipes, além de desenvolver habilidades técnicas, interpessoais e comunicativas ^19^. A convivência entre residentes e profissionais favorece também a superação de práticas centradas no modelo biomédico, aproximando o cuidado das diretrizes da atenção integral ^22^.

Avaliou-se a percepção de usuários da atenção primária à saúde com a ferramenta Primary Care Assessment Tool e identificou-se que a presença de programas de residência, especialmente em medicina de família e comunidade, impactou positivamente a avaliação dos serviços ^16^. Esse fator elevou a pontuação geral de qualidade atribuída pelos usuários ^16^.

No contexto hospitalar, residentes de enfermagem relataram avanços clínicos, olhar crítico e maior sensibilidade às necessidades dos usuários ^19^. A troca de saberes entre residentes e equipes tem viabilizado modelos de cuidado mais integrados, alinhados às diretrizes do SUS e à realidade da atenção primária à saúde ^22^. A presença dos residentes contribui para repensar práticas centradas na doença e orientadas por condutas fragmentadas, o que promove abordagens mais amplas e centradas na pessoa ^22^.

As boas práticas em saúde decorrem da colaboração interprofissional e da integração entre equipes, configurando-se como um dos principais efeitos indiretos da atuação dos residentes. Observou-se que a presença desses profissionais impulsionou a comunicação e a cooperação entre anestesiologistas, cirurgiões e equipes de enfermagem, o que promoveu decisões compartilhadas e reduziu conflitos nas salas de cirurgia ^17^. 

A vivência no SUS contribuiu para desconstruir preconceitos sobre a atuação na atenção primária à saúde ^17^^,^^22^. A residência, ao integrar ensino e assistência, estimula a formação crítica e reflexiva sobre a realidade do SUS, o que favorece a formação de profissionais com pensamento crítico, competência técnica e compromisso com a transformação do sistema ^21^^,^^24^.

A experiência hospitalar também se mostrou decisiva para o desenvolvimento de habilidades técnicas e de julgamento clínico. A imersão na assistência durante esse período proporcionou aos residentes melhor compreensão dos agravos, além do aprimoramento de competências essenciais, como a tomada de decisões ^19^. Esse processo de aprendizagem contínua é fundamental para a formação de profissionais aptos a enfrentar os desafios da saúde pública ^19^.

## Discussão 

Os resultados desta revisão apontaram para o papel estratégico dos programas de residências em saúde na qualificação da assistência prestada no SUS, especialmente ao impulsionar práticas colaborativas, fomentar a reorganização dos processos de trabalho e contribuir para a resposta aos agravos nos diferentes níveis de atenção. Observou-se que os programas de residência atuam como importantes indutores da resolutividade dos serviços, do fortalecimento de vínculos com a comunidade, da revisão de práticas clínicas e da promoção de abordagens interprofissionais. Isso favorece a integração entre as equipes e influencia, de maneira positiva, os fluxos assistenciais ^26^. 

Apesar dos avanços identificados, persistem desafios importantes. Esta revisão incluiu estudos publicados entre 2012 e 2024 com diversidade de delineamentos e populações e evidenciou uma lacuna significativa na mensuração dos efeitos sistêmicos das residências. Observou-se a predominância de abordagens qualitativas, em geral centradas em recortes locais, análises pedagógicas ou relatos de experiência de indivíduos vinculados ou que já tiveram vínculo com os programas (residentes, preceptores, tutores ou docentes). 

Embora esses trabalhos tenham contribuído para documentar vivências, contextos de prática e processos formativos, ofereceram alcance limitado para extrapolações e não avançaram para a avaliação mais ampla dos efeitos das residências no sistema de saúde. Assim, persiste na literatura uma lacuna de estudos primários com maior rigor metodológico, capazes de investigar de forma sistemática e abrangente as contribuições desses programas para os serviços e as redes de atenção.

Por se tratar de revisão de escopo rápida, devem-se considerar as limitações metodológicas inerentes ao delineamento, como o uso de atalhos na seleção dos estudos e na extração dos dados, realizadas de forma não cegada e não pareada, além da não realização de busca manual para identificar estudos potencialmente elegíveis. Tais procedimentos, ainda que previamente calibrados pela equipe, não eliminam o risco de viés de seleção e análise.

Em muitos contextos, a qualificação da assistência e a reorganização dos processos de trabalho observadas durante a vigência da residência tendem a se dissipar após a saída do residente do cenário. Isso indica fragilidade na institucionalização das práticas e na consolidação de mudanças estruturais ^27^. Em diversas situações, os residentes acabam sendo alocados para suprir demandas dos serviços de saúde e assumem atribuições que, por vezes, vão além do esperado para sua etapa de formação profissional ^28^. Essa realidade pode enfraquecer o caráter formativo da residência ^29^.

Muitos residentes relataram que enfrentaram desafios como cargas horárias intensas, acúmulo de atividades assistenciais, reconhecimento ainda limitado no processo de trabalho e dificuldades nas relações interpessoais nas equipes. Também se destacaram as incertezas quanto à inserção no mercado de trabalho após a residência ^28^^,^^29^. Tal contexto amplia os debates sobre saúde mental e os caminhos profissionais dos egressos ^30^.

Esses desafios coexistem com efeitos positivos relevantes identificados nos programas de residência, que contribuem para o fortalecimento dos serviços e as redes de atenção do SUS. Destacam-se a ampliação do acesso, a redução de internações evitáveis, o fortalecimento da resolutividade da atenção primária à saúde, a melhoria da qualidade do cuidado e o estímulo a práticas colaborativas e interprofissionais. Também contribuem para a reorganização dos processos de trabalho, a inovação assistencial, o fortalecimento de vínculos com os usuários e a integração entre ensino e serviço. 

No entanto, consolidar as residências como estratégias estruturantes do SUS exige reconhecer as tensões que atravessam sua implementação, bem como os limites impostos por modelos institucionais que nem sempre estão preparados para sustentar os pressupostos pedagógicos e os compromissos éticos e políticos desses programas. Consolidar as residências no SUS exige ampliar vagas, valorizar a preceptoria, qualificar os cenários de prática e o reconhecimento do residente como sujeito ativo em formação.

## Data Availability

Foi criado o repositório online com as planilhas eletrônicas de seleção de textos completos e extração de dados, disponível em: https://drive.google.com/drive/folders/1OfOk5TILreYuu2D30OiY-hcRexT8XNIT?usp=sharing. De forma complementar, os dados também foram disponibilizados no projeto da Open Science Framework (doi.org/10.17605/OSF.IO/JZH3X).

## Material Suplementar

Tabela Suplementar 1

Tabela Suplementar 2

Tabela Suplementar 3
